# INTENTIONAL SUPPORT REDUCES TECHNICAL BARRIERS TO BEHAVIORAL INTERVENTIONS FOR DEMENTIA CAREGIVERS

**DOI:** 10.1093/geroni/igae098.2980

**Published:** 2024-12-31

**Authors:** Emily Tupper, Stephanie Irish, Sarah Gothard, Christina Zonker, Aimee Mooney, Allison Lindauer

**Affiliations:** Oregon Alzheimer’s Disease Research Center, Oregon Health & Science University, Portland, Oregon, United States; Oregon Alzheimer’s Disease Research Center, Oregon Health & Science University, Portland, Oregon, United States; Oregon Alzheimer’s Disease Research Center, Oregon Health & Science University, Portland, Oregon, United States; Oregon Alzheimer’s Disease Research Center, Oregon Health & Science University, Portland, Oregon, United States; Oregon Health & Science University, Portland, Oregon, United States; Oregon Alzheimer’s Disease Research Center, Oregon Health & Science University, Portland, Oregon, United States

## Abstract

Utilizing technology to provide research interventions can reduce historic barriers to participation, such as travel for study visits, but also presents new challenges for participant retention. Technological issues, including malfunctioning equipment or operator error, are routinely identified as a source of attrition in telehealth studies, and are often associated with a lack of technical support. The Tele-STELLA study, a telehealth intervention aimed at supporting caregivers of persons with dementia, found employment of a tech-support staff eliminated technological issues as a source of attrition. In addition to an interventionist leading each session, a support person was assigned to monitor and address any technical issues. Technical difficulties and subsequent support were recorded for each session. Of 374 recorded sessions, interventionists reported 64(17%) technical difficulties, of which 62(16.5%) were resolved in less than 15 minutes, allowing the session to continue as scheduled; 2(0.5%) sessions required rescheduling. Participant feedback about overall technical experience was gathered at 8 and 16 weeks, and at the time of withdraw. Of 150 enrolled participants, 49 withdrew before completing the study(33%), and experience data was available for 30(61%). Of those, none reported technical issues as a reason for withdrawing, and 28 reported to “strongly agree” or “agree” that they had good technical support(93%). Although attrition is higher than those reported in comparable studies, Tele-STELLA circumvented the barrier of technological issues with targeted technical support during sessions. These findings suggest that purposeful technical support could eliminate a known cause for similar interventions.

